# Impulse dispersion of aerosols during playing the recorder and evaluation of safety measures

**DOI:** 10.1371/journal.pone.0266991

**Published:** 2022-09-26

**Authors:** Marie Köberlein, Laila Hermann, Sophia Gantner, Bogac Tur, Gregor Peters, Caroline Westphalen, Tobias Benthaus, Michael Döllinger, Stefan Kniesburges, Matthias Echternach

**Affiliations:** 1 Department of Otorhinolaryngology, Division of Phoniatrics and Pediatric Audiology, University Hospital Ludwig-Maximilians Universität, Munich, Germany; 2 Department of Otorhinolaryngology, Head and Neck Surgery, Division of Phoniatrics and Pediatric Audiology, University Hospital Friedrich-Alexander-Universität, Erlangen, Germany; 3 Institute and Clinic for Occupational, Social and Environmental Medicine, University Hospital Ludwig-Maximilians-University, Munich, Germany; Shahrood University of Medical Sciences, ISLAMIC REPUBLIC OF IRAN

## Abstract

**Introduction:**

Group musical activities using wind instruments have been restricted during the CoVID19 pandemic due to suspected higher risk of virus transmission. It was presumed that the aerosols exhaled through the tubes while playing would be ejected over larger distances and spread into the room due to jet stream effects. In particular, the soprano recorder is widely used as an instrument in school classes, for beginners of all age groups in their musical education, in the context of leisure activities and in professional concert performances. Understanding the aerosol impulse dispersion characteristics of playing the soprano recorder could assist with the establishment of concepts for safe music-making.

**Methods:**

Five adult professionally trained soprano recorder players (4 female, 1 male) played four bars of the main theme of L. van Beethoven’s “Ode to Joy” in low and in high octaves, as well as with 3 different potential protection devices in the high octave. For comparison they spoke the corresponding text by F. Schiller. Before each task, they inhaled .5 L of vapor from an e-cigarette filled with base liquid. The vapor cloud escaping during speaking or playing was recorded by cameras and its spread was measured as a function of time in the three spatial dimensions. The potential safety devices were rated for practicability with a questionnaire, and their influence on the sound was compared, generating a long-term average spectrum from the audio data.

**Results:**

When playing in the high octave, at the end of the task the clouds showed a median distance of 1.06 m to the front and .57 m diameter laterally (maxima: x: 1.35 m and y: .97 m). It was found that the clouds’ expansion values in playing the recorder with and without safety measures are mostly lower when compared to the ordinary, raised speaking voice of the same subjects. The safety devices which covered the instrument did not show clear advantages and were rated as unpractical by the subjects. The most effective reduction of the cloud was reached when playing into a suction funnel.

**Conclusion:**

The aerosol dispersion characteristics of soprano recorders seem comparable to clarinets. The tested safety devices which covered holes of the instrument did not show clear benefits.

## Introduction

Research in recent years has focused on how the SARS-CoV-2 virus spreads via exhaled airborne particles [1, 2]. Virus-loaded particles are caused by movements of the mucous membranes of the respiratory tract, which tear up the secretion and saliva in the airways. The smaller particles (<5 μm), so-called aerosols, can remain airborne for several hours, possibly travelling, accumulating and being inhaled by other individuals. In this regard, musical activities such as choir singing or wind instrument playing have been suspected of having higher virus transmission risks due to their inherent increased exhalation activity [3, 4], and have therefore been restricted by laws in many countries around the world. The combination of jet stream effects, accumulation of exhaled particles, and group gatherings led to the closing of concert halls, as well as the prohibition of musical events, rehearsals, and in-person music lessons in schools [5]. Therefore, institutions in which playing a wind instrument plays an important role, such as theatres, concert halls, schools, music schools and music universities had to invent safety concepts to be able to maintain offering of musical activities. These concepts often included the frequent disinfection of the playing spaces, frequent aeration, distancing or even teaching online, as well as covering the instruments with masks or fabrics [6–14]. In order to provide helpful information for the development of such concepts, several studies tried to figure out if wind instruments were increasing the infection risks due to both aerosol generation and dispersion, or if the instruments were decreasing the risks by capturing the condensed aerosols inside the instruments: While a study on vuvuzelas, which are straight narrow tubes comparable to recorders, showed the ejection of much higher amounts of aerosols than in shouting [15], a more recent study found smaller aerosol expulsion from classical wind instruments compared to vocalization [16]. A study by He et al. [17] divided various orchestral wind instruments into different risk categories by their aerosol emissions. They found flutes and clarinets, which resemble the recorder in mechanism or shape, respectively, to be only at intermediate transmission risk, while trumpet and oboe belonged to the high-risk category. In contrast, a study analyzing the impulse dispersion of aerosols ranked the flute to be more risky than clarinet and trumpet, due to the larger distances reached by the aerosols [18]. A study by Stockman et al. [19] found comparable amounts of airborne particles in clarinets and singing, as well as effective reduction of aerosol concentration and dispersion by surgical masks. Nevertheless, their results showed high unsteadiness and situation-dependent variations in the concentrations and dispersions of the emitted vapor clouds. In agreement with this, Wang et al. [20] assessed woodwind instruments, i. e., clarinets and flutes, to be high risk instruments. They measured source aerosol concentrations and source aerosol emission rates, and found them to be reduced by bell covers.

All these studies concentrated on a variety of orchestral instruments, while the recorder, which is a common instrument for musical education and leisure activity in children and adult amateurs as well as professional musicians, has, to the best of the authors’ knowledge, not been addressed. It could be speculated that the results of other studies cannot be transferred simply. Referring to a representative study on amateur music in Germany, the recorder is played by 22% of female and 3% of male amateur musicians aged 16+. In children aged 6–15, 96% play an instrument, with the recorder found in second place by popularity, being played by 24% of these children [21]. The soprano recorder is, furthermore, used in music classes of public schools all over the world owing to its low purchase costs and its ease of use. Consequently, the actual number of children using recorders can be assumed to be much higher.

In order to help facilitate the transmission risk management for respiratory infectious diseases and find suitable measures for recorder classes, the presented study investigates the properties of the impulse dispersion of aerosol clouds emitted during playing soprano recorders. It also evaluates several protective devices with the expectation of decreasing the radius of aerosol dispersion by covering holes or suction. The main aim of the study is to fill in gaps in knowledge in this area.

## Material and methods

### Subjects

After the approval of the local ethical committee (20–1065), five full-time student recorder players from the local University of Music (4 female, 1 male) were recruited. None of the subjects showed respiratory problems in their respective medical histories and spirometry (ZAN, Inspire Healthcare, Oberthulba, Germany). There were no signs of acute infection based on their answers to an acute CoVId19 infection questionnaire, which covered the symptoms of fever, cough, runny nose, shortness of breath, sore throat, headache, body aches, smell and taste disorders. Furthermore, the questionnaire asked for the CoVID19 test result and possible contact with infected persons in the previous 14 days.

### Tasks

**Fig 1:**
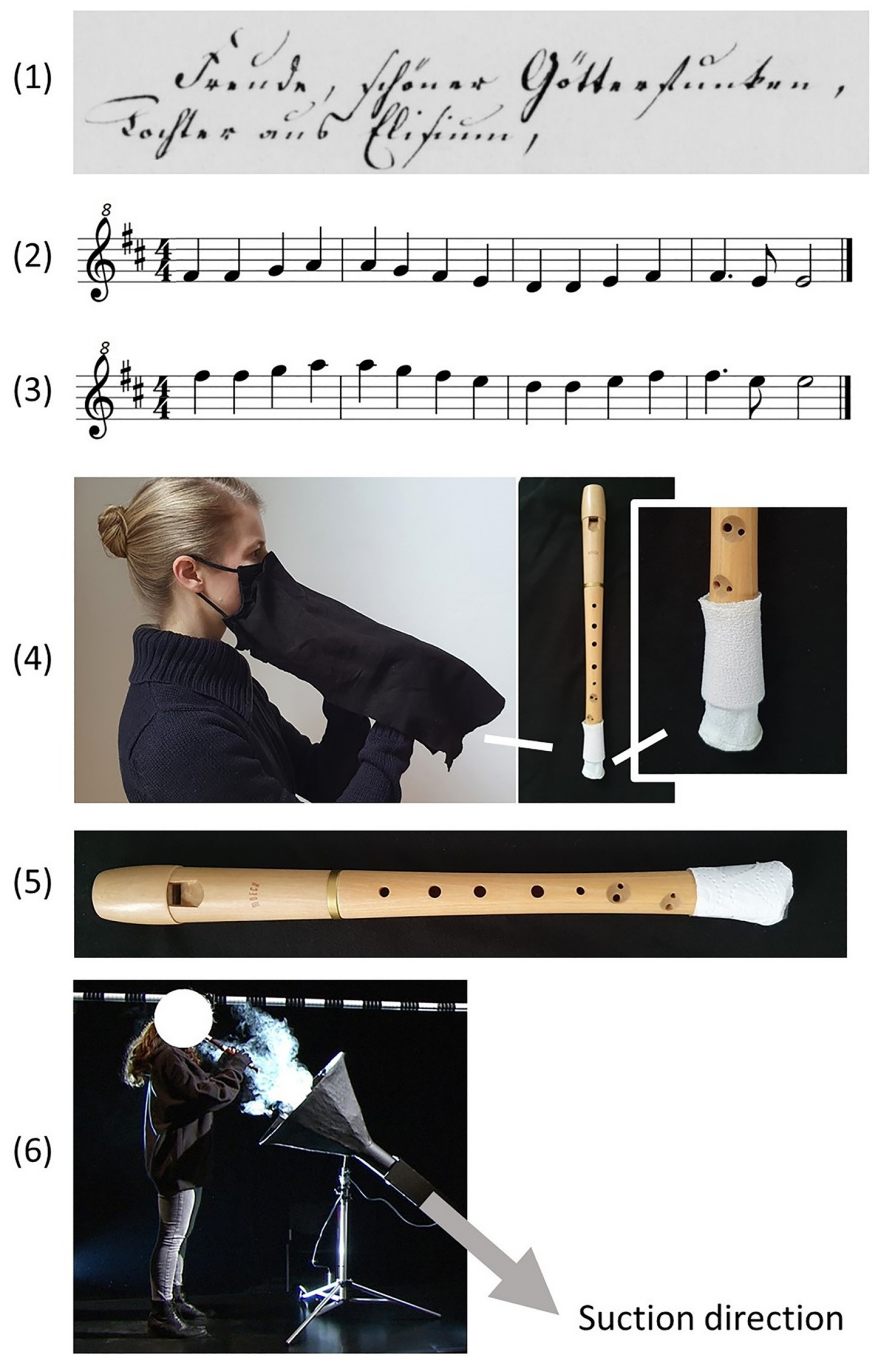
All performed tasks. (1) Reciting the excerpt from Friedrich Schiller’s “Ode to Joy” [23]. (2) and (3) playing the melody. The potential protection devices: (4) cloth mask and bell hole cover, (5) paper towel, (6) suction funnel.

In the experiment, the subjects performed six tasks, see also Fig 1. The whole series of tasks was performed once by each subject and in the same order:

1. Reciting the sentence “Freude schöner Götterfunken, Tochter aus Elisium” (“Ode to joy” by Friedrich Schiller (used also by Beethoven in his ninth symphony, 4^th^ movement)) with raised voice.
2. Loudly playing four bars of the melody of the “Ode to joy” by Ludwig van Beethoven in their lower octave, starting from F#5 (fundamental frequency (*f*_*o*_) approx. 740 Hz). The speed was set as approx. 120 bpm.
3. Loudly playing the same melody in their higher octave, starting from F#6 (*f*_*o*_ approx. 1475 Hz).
4. Playing the same melody in their higher octave with a specially designed extended cloth-mask that covered the player’s mouth and nose and the entire instrument (material: 200g/m2 cotton molton), as well as a cover tissue for the bell hole called “Plopp-Schutz” (©Bonner Textilmanufaktur [22]). The cloth extension lay over the whole instrument, including the sound hole, and was not closed at the bottom, so that the players could reach the instrument with their hands. The extension was sewn to a conventional cloth mask with an aperture at the mouth level so that the mouthpiece of the recorder could be reached by the player.
5. Playing the melody in the higher octave, the bell hole covered with household paper towel, as suggested by Bauhaus-Universität Weimar [7]. The paper towel was attached to the foot of the instruments with conventional tape.
6. Playing the above-mentioned melody in the higher octave into a suction funnel (diameter at the inlet: .6 m, diameter at the outlet: .1 m, length .45 m, extended by a pipe of 1 m. Construction details: The supporting structure / substructure consists of 3 mm welding wire. The wires are soldered together. On top of that are several layers of newspaper soaked in paste. After hardening, several layers of glue-soaked packing paper were put over it. The extraction took place via a 100 mm tubular motor, which is also used in extractor hoods.).

At the time of the measurements no safety devices for recorders other than the “Plopp-Schutz” were on the market. The devices represented in this study were derived from concepts for other instruments found in online research. A selection criterion was the feasibility of replication, should it prove effective.

### Test setup

Each task lasted approximately 8 seconds. Before the subjects started the tasks, they inhaled .5 l vapor from the base liquid through a Lynden Vox e-cigarette (Lynden GmbH). The base liquid consists of 50% glycerin and 50% polypropylene glycol. The size of the aerosols in the e-cigarette vapor is in the range of 250–450 nm [24], which lies within the size range of potentially virusladen aerosols generated by human exhalation [1, 25, 26]. A spirometer (ZAN, Inspire Healthcare, Oberthulba, Germany) was mounted on top of the e-cigarette to measure the volume of the vapor inhaled by the subject.

The subjects were standing on a marked point on a stage while inhaling and performing. They were asked to avoid any motion after finishing the tasks, in order to avoid convectional flows. As in previous experiments [18, 27–31], the measurements were conducted in a Bavarian Television Broadcasting Studio with the dimensions of 27 m x 22 m x 9 m. Three synchronized high-definition television cameras (Sony, Tokio, Japan, resolution 1920 × 1080 pixels, 25 fps) recorded the tasks from a side view (C1), a front view (C2) and a top view (C3). The studio was aerated before and after each task via opposing doors opened for a minimum of 2 minutes. The room had a constant humidity of 29.8% and a constant temperature of 21.0°C. Three spotlights illuminated the white aerosol clouds against the black studio walls. For the conversion of pixels into metric values in the data processing, 3 metric scaling bars were installed at the performance stage to cover all 3 spatial dimensions.

### Data analysis

**Fig 2:**
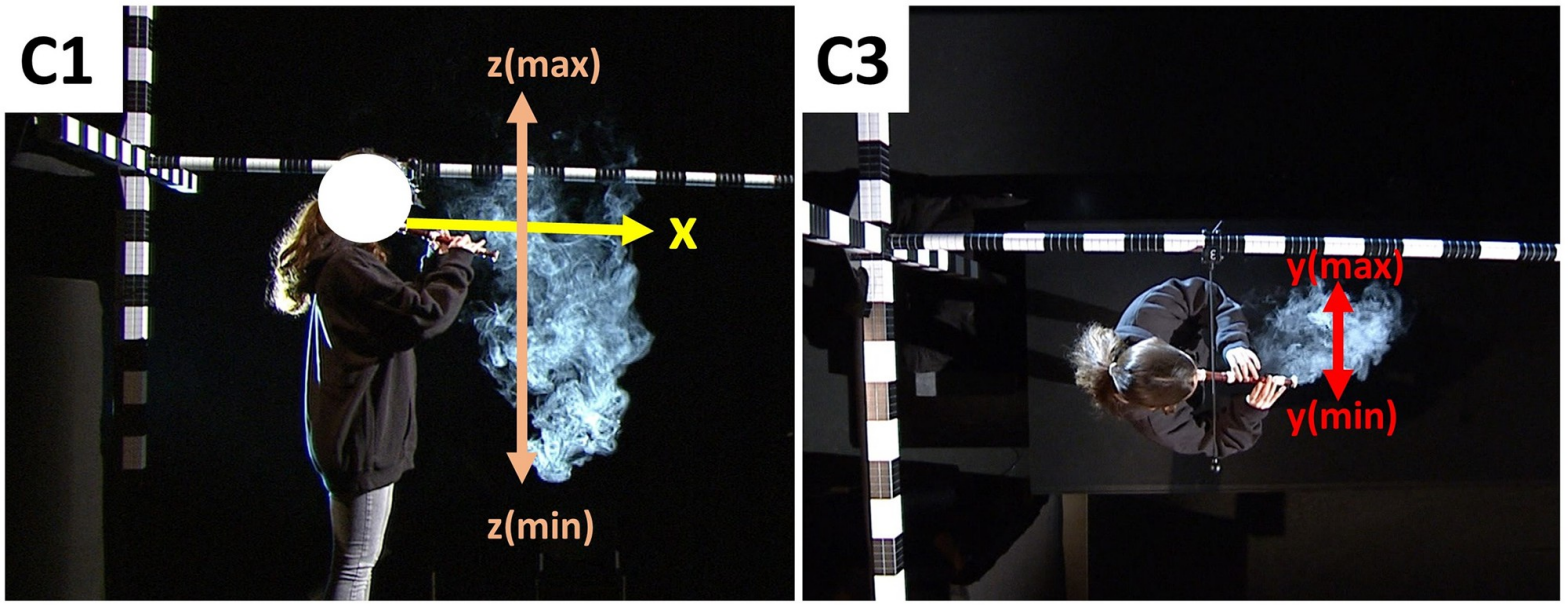
Display of the camera perspectives. Pictures of camera C1 (left) recording the x-dimension to the front and the vertical z-dimension from a side view, and camera C3 (right) recording the lateral y-dimension from a top view.

Similar to previous experiments [18, 27–31], the video footage was converted into negative black and white. The subjects were asked to wear black clothes. However, some uncovered parts of the human body like hands or faces were masked using the digital image editing software Sensarea (Grenoble Institute of Technology (INPG), France) to avoid segmentation errors. The expansions of the expelled aerosol clouds were segmented in each video frame using the software Glottis Analysis Tools (University Erlangen-Nürnberg, Germany) [32]. The footage of C2 had to be excluded from further analysis due to light reflections which led to artifacts in the segmentation process. The footage of C1 was used to measure the maximum expansion to the subjects’ front (x-direction) and in maximum positive and negative vertical direction (z-direction). The footage of C3 provided the side expansion of the aerosol clouds, see Fig 2. The position of the mouth was set as starting point of the aerosol cloud dispersion.

For the temporal representation of the clouds’ dispersion, the time point zero was defined as the end of a task (0 point in the time domain), i.e., where phonation or playing stopped. Consequently, the start of the task had negative values in the time domain. The temporal devolution of the maximum dispersion in each direction was filtered by a moving median filter (window length of 30 time points) for each player and subsequently smoothed by a cubic spline interpolation in order to remove segmentation outliers, both steps carried out in Matlab (The Mathworks Inc., Natick, MA). In task 6 (suction funnel), the pipe’s end was not connected to a longer tube which would guide the vapor outside of the room so that the vapor was released into the studio. However, this released cloud was not considered in the segmentation as it was clearly distinguishable from the clouds directly originating from the player and/or the recorder.

### Acoustical evaluation of the potential safety devices

Additionally, the subjects were asked to perform the melody of task 3 without e-cigarette vapor but in 30 cm distance to a D:SCREET 4061 microphone (DPA Microphones A/S, Alleroed, Denmark). The microphone was connected to the computer via a Scarlett 2i2 audio interface (Focusrite Plc, High Wycombe, England). The audio signal was then sampled with the software Audacity26 (MuseCY SM Ltd, Limassol, Cyprus) at a sampling rate of 44.1 kHz. The melody was performed (1) with each of the potential safety devices and (2) without any. From the audio recordings, Long Term Average Spectra (LTAS) were obtained using the software Wavesurfer (version 1.8.8, Centre for Speech Technology, KTH Stockholm; settings: FFT: Hanning, 512 points, range: -110 dB), in order to detect changes in the spectral characteristic of the instrument’s sound caused by the safety devices.

### Subjective evaluation of the potential safety devices

The subjects also answered a questionnaire consisting of visual analogue scales (0–100 mm) with statements on the freedom of movement, sound attenuation, inhalation sufficiency, and concert practicability to subjectively evaluate the application of the analyzed safety devices, see S1 File.

### Statistical analysis

Due to the small number of subjects, statistical calculations were considered not meaningful.

## Results

All subjects were able to perform all tasks without any dropout. The vapor escaped the instrument mainly through the labium and the bell hole and merged into one cloud.

At the end of the speaking task, the maximum expansion of the aerosol clouds reached 1.32 m to the front, with a median of .83 m. The dispersion at the end of the task for playing the recorder in the low and the high octave exhibited maximum values comparable to speaking, i. e., in a range of ± .1 m, with slightly higher median values to the front (T2: 0.97 m; T3: 1.06 m), see Table 1.

**Table 1:** The maximum and median values for all performed tasks.

		x-Dimension			Diameter y-Dimension		
**T1 Speaking**	Timepoint	0[s]	3[s]	10[s]	0[s]	3[s]	10[s]
	Median	0.83 m	1.12 m	1.41 m	0.84 m	0.97 m	0.58 m
	Maximum	1.32 m	1.89 m	2.58 m	1.16 m	1.43 m	1.62 m
**T2 Playing Low**	Timepoint	0[s]	3[s]	10[s]	0[s]	3[s]	10[s]
	Median	*0*.*97 m*	**0.90 m**	**0.97 m**	**0.47 m**	**0.42 m**	**0.04 m**
	Maximum	1.38 m	**1.44 m**	**1.50 m**	**0.77 m**	**1.00 m**	**1.14 m**
**T3 Playing High**	Timepoint	0[s]	3[s]	10[s]	0[s]	3[s]	10[s]
	Median	*1*.*06 m*	**0.93 m**	**1.01 m**	**0.57 m**	**0.53 m**	**0.23 m**
	Maximum	1.35 m	**1.62 m**	**2.17 m**	**0.97 m**	**0.83 m**	**0.72 m**
**T4 Mask & Bell Hole Tissue**	Timepoint	0[s]	3[s]	10[s]	0[s]	3[s]	10[s]
	Median	**0.65 m**	**0.81 m**	**0.70 m**	0.56 m	*0*.*67 m*	*0*.*65 m*
	Maximum	1.40 m	*1*.*74 m*	*2*.*28 m*	**0.78 m**	0.77 m	0.81 m
**T5 Paper Towel**	Timepoint	0[s]	3[s]	10[s]	0[s]	3[s]	10[s]
	Median	*1*.*17 m*	*1*.*28 m*	*1*.*36 m*	0.59 m	*0*.*70 m*	*0*.*70 m*
	Maximum	1.31 m	**1.51 m**	**1.93 m**	**0.74 m**	*0*.*96 m*	*1*.*29 m*
**T6 Suction Funnel**	Timepoint	0[s]	3[s]	10[s]	0[s]	3[s]	10[s]
	Median	**0.65 m**	**0.42 m**	**0.39 m**	**0.19 m**	**0.08 m**	**0.08 m**
	Maximum	**1.16 m**	**1.14 m**	**1.46 m**	**0.65 m**	**0.55 m**	**0.62 m**

Table 1 lists the maximum and median values at the time points 0 s (end of the task), as well as 3 s and 10 s after the end of the task. Italic values in T2-3 show a minimum of .1 m higher value in comparison to T1. Bold values are more than .1 m below the compared value from T1. T4-6 are compared to T3: Bold values are at least .1 m below the compared value of T3, italic markings show a minimum of .1 m higher value than in T3. Unmarked values are within a ± .1 m range of the compared value.

The lateral expansion of the clouds from speaking at the end of the task showed a maximum diameter of 1.16 m with a median of .84 m. All values of the aerosol clouds’ lateral expansions during playing the recorder in high and low octave were lower than in speaking (Table 1).

At the time points 3 and 10 seconds after the end of the task, the clouds’ expansions from playing the recorder were found below all compared values of the speaking task. A maximum expansion to the front of 2.17 m was reached by one subject 10 seconds after playing in the high octave, which is still .41 m below the maximum value 10 seconds after speaking (2.58m).

A maximum lateral expansion diameter of 1.14 m was reached 10 seconds after playing in the low octave, which is .48 m below the maximum value 10 seconds after speaking (1.62 m).

**Fig 3:**
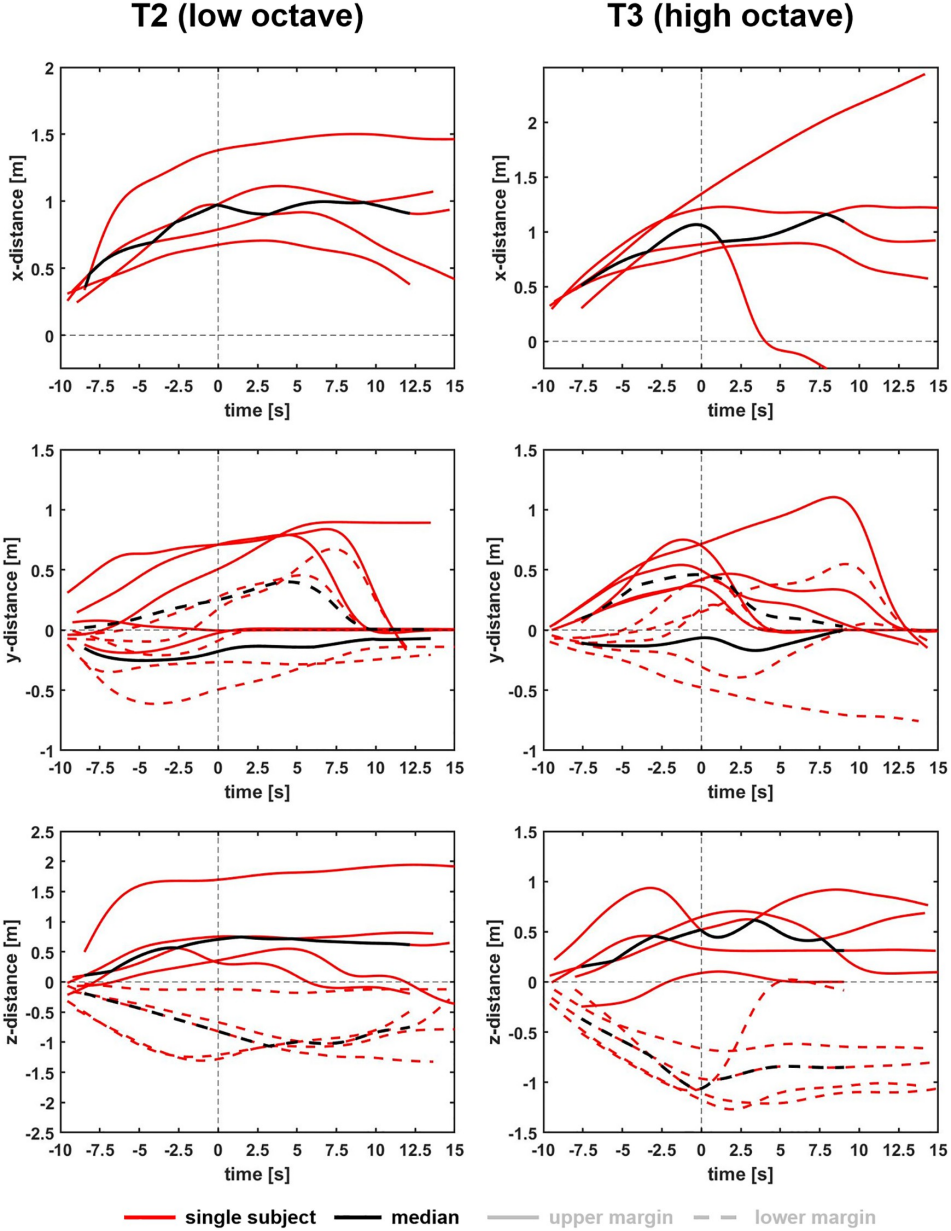
Results of tasks 2 and 3. Aerosol dispersion distances of all subjects (red) and medians (black) over all subjects in task 2 (low octave) and task 3 (high octave) for x- (front), y- (side), and z- (vertical) direction. The distances are measured from the subjects’ mouth. The zero in the time domain is set as the end of the task.

Fig 3 shows the dispersion for all subjects’ tasks 2 and 3 and the corresponding medians.

All safety devices influenced the dispersion patterns during playing the recorder when compared to task 3, i. e., playing in the high octave without safety device.

When playing with the mask and bell hole cover (T4), the median in x-direction to the front at the end of the task was .41 m below the compared value for task 3 and stayed below the median values for the later time points. The maximum value to the front at the end of the task was comparable to T3 and 10 seconds later the maximum exceeded the compared value of T3 by .11 m.

With regard to the lateral expansion at the end of the task, the median diameters of T3 and T4 were comparable. The maximum value for T4 at the end of the task was .19 m lower than in T3 (Table 1).

For task 5, i. e., applying the paper towel during playing, the median values to the front were higher than in T3, while the maxima were below the compared values, but only .04 m lower at the end of the task. In the y-dimension at the end of the task, the median value was comparable to the value of T3, with a maximum of .23 m below the value of T3. All lateral values at later time points were higher than in the compared task T3, reaching a largest diameter of 1.29 m after 10 seconds.

All listed values in task 6 with the suction funnel were below the values of T3 (Table 1).

**Fig 4:**
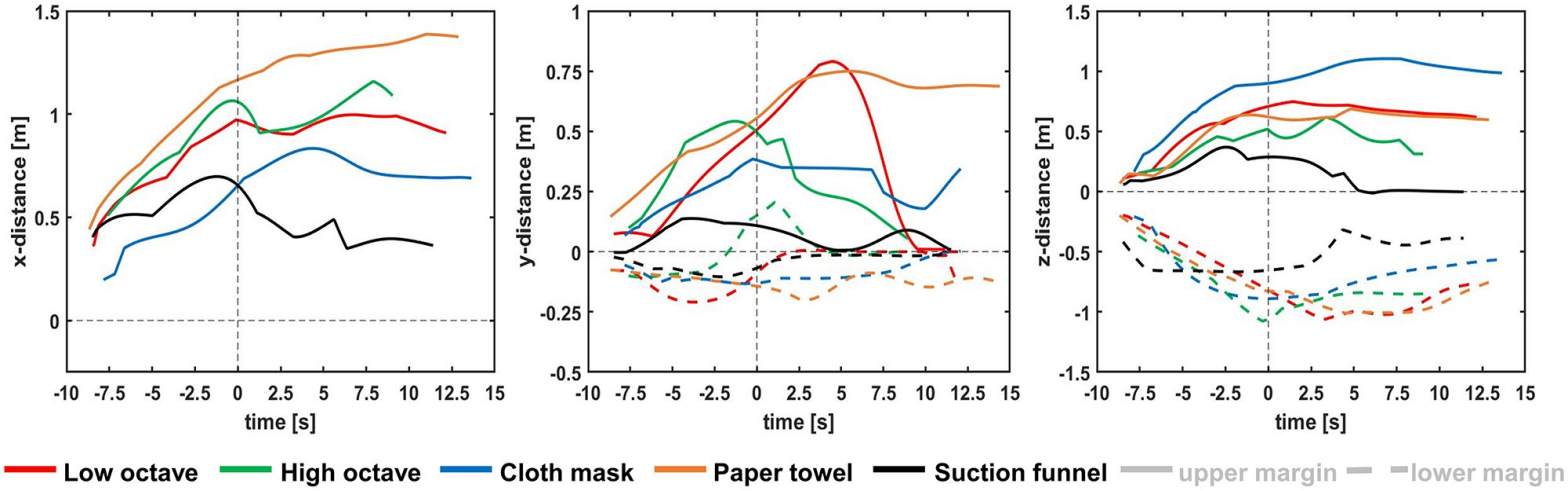
Development of the medians of all three dimensions over time. The zero-point in the time domain marks the end of the phonation task. The distances are measured from the subjects’ mouths.

Fig 4 provides the medians for the safety devices in comparison to normal playing in low and high octave. Regarding the vertical direction, playing in the low octave reached the highest maxima upwards from the subject’s mouth with 1.7 m at the end of the task and 1.91 m at time point 10 s.

The highest median values of all tasks in positive z-direction (upwards) were reached when playing with mask and bell hole tissue, showing .9 m at the end of the task and 1.04 m at time point 10 s.

### Evaluation of safety devices

**Fig 5:**
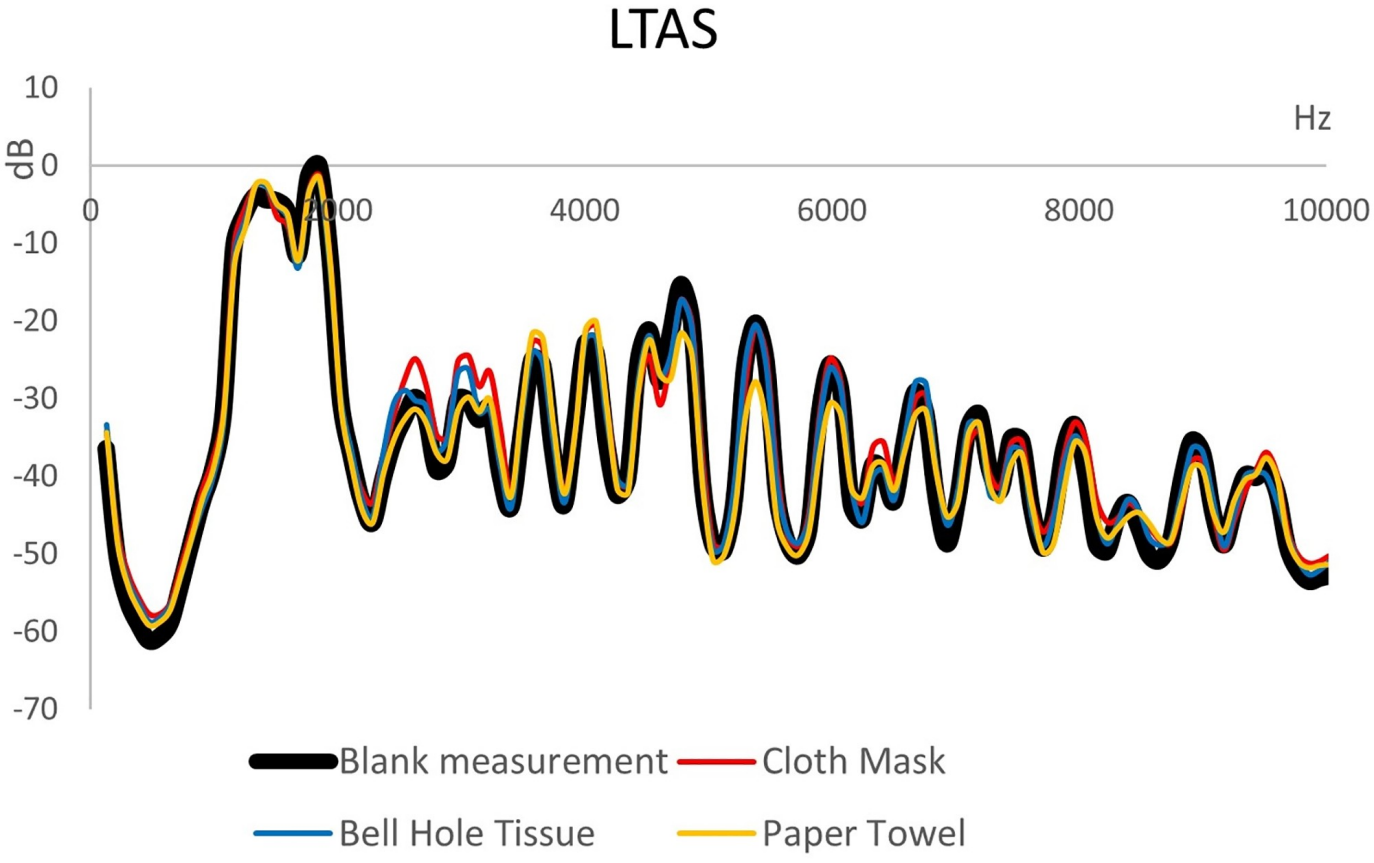
Long-term average spectra (LTAS) when playing the same melody with and without potential safety devices. The presented curves are the averages of all players sorted by task.

The long-term average spectrum of playing in the high octave without device (pure) and with the mask or tissue or paper towel is depicted in Fig 5. No significant attenuations or deviations were observed. The mean squared errors are 4.7 for the extended mask, 2.77 for the bell hole tissue and 5 for the paper towel.

**Fig 6:**
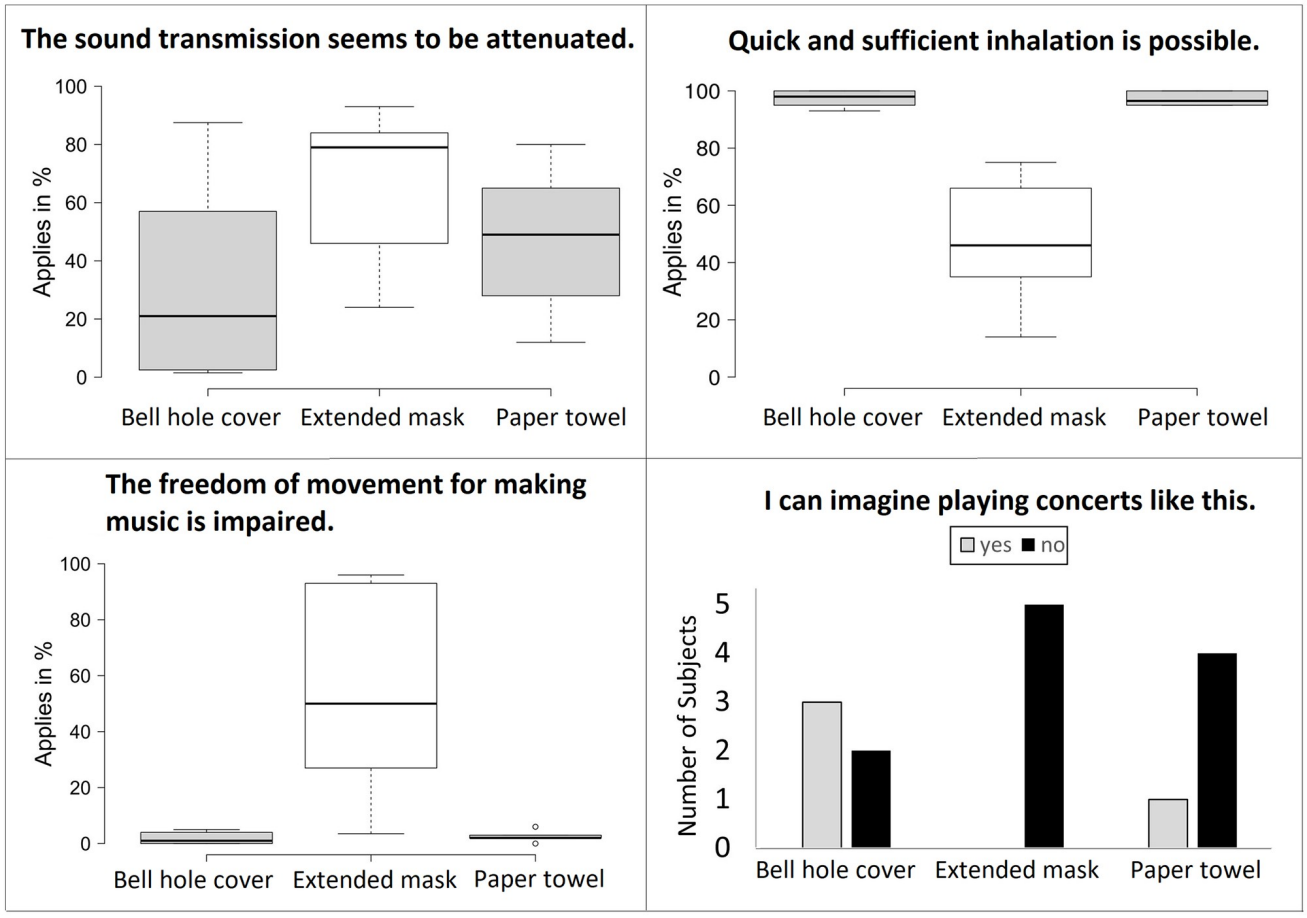
The subjects’ evaluation of the practicability of the potential safety procedures. The results were obtained from a questionnaire (see S1 File) with visual analogue scales.

Fig 6 shows the results of the evaluation questionnaires concerning mask, tissue, and paper towel. The perception of sound attenuation was reported for all of the safety devices, but was the least for the bell hole tissue. For the paper towel, it was shown that the covering of the bell hole prohibited the production of low pitches where all fingers close their assigned hole. Especially for the mask, the musicians reported problems in sufficient inhalation and restriction of movement. All the subjects rejected the application of masks for use in concerts. All except one musician rejected the paper towel. The tissue was rated as practicable in concerts by three of the five subjects.

## Discussion

This study is the first to gather information on the aerosol characteristics of recorders and it investigates the impulse dispersion of aerosols during playing the soprano recorder alone and with three potential safety devices. In general, it was found that the impulse dispersion characteristics were comparable to those of clarinets [18] but not of flutes, and that a suction funnel can reduce the aerosol dispersion significantly, while other devices covering the instrument were problematic concerning the aerosol dispersion and/or practical implementation.

Group musical activities were restricted in many countries during the Covid19 pandemic, due to the estimated higher risk of virus transmission in playing wind instruments [3, 4]. The risk is assumed to be higher due to jet streams through narrow instruments, as well as the accumulation and suspension of aerosols in rooms where groups are gathered.

Aerosol emission rates and impulse dispersion of several orchestra instruments have already been investigated in previous studies [15–20], but none of these studies included the recorder. However, the recorder is a very common instrument, not only in professional settings but also in music education and amateur music-making [21]; understanding the dispersion characteristics could help in risk management and adjustment of safety concepts.

When playing in the high octave, at the end of the task, the clouds showed medians of 1.06 m to the front (x-direction) and .57 m diameter laterally (y-direction), with maxima of x: 1.35 m and y: .97 m. Compared to the dispersion values of other instruments in a previous study with the same experimental setup [18], without safety devices at the end of the task, the straight-built recorder produced larger distances than the winding-built trumpet (the trumpet values were medians: x: .86 m and y: .3 m; maxima: x: 1.20 m and y: .52 m). Even though the mechanism of the recorder is somewhat similar to the flute, dividing the airflow from the windway above the block at the labium, the generated jet stream of aerosols did not go as far as for the flutes (flutes’ medians: x: 1.51 m, y: .64 m; maxima: x: 1.88 m, y: 1.23 m). The values of the recorder were rather comparable to the shape-resembling clarinet (medians x: 1 m, y: .55 m; maxima x: 1.51 m, y: .95 m).

Similar to the clarinets in the previous study [18], the clouds’ frontal expansions of the recorders generally stagnated or decreased after the end of the task at a distance of around 1 m. For a single subject the cloud continued to expand and reached a maximum distance of 2.17 m to the front, 10 seconds after playing in the high octave, which exceeds former recommendations of a safety distance of 2 m to the front for wind instruments in general. Accordingly, the lateral expansions decreased after the end of the task and increased for one subject after playing in the low octave, reaching a maximum diameter of 1.14 m 10 seconds after the end of the task. Considering the individually different expansion dimensions of each cloud after the end of the task (see for example Fig 3: T3 in x-direction), it seems likely that, after the primary impulse, multifactorial influences such as convectional flows or temperature differences make the diffusion of the clouds unreliable to calculate and therefore questionable.

It must be taken into account that this study measured only trained, adult recorder players playing loudly; playing at moderate volume was not examined, and children and amateurs might have less exhalation power and playing at moderate volume was not examined. These factors might influence the distance of the impulse dispersion. As observed by Volckens et al. [33] and Firle et al. [34], the inter-individual deviations and within-person variabilities in aerosol emission while playing wind instruments can be expected to be high. This might be due to differences in lung volumes [35], sound pressure levels [17, 36] and specific saliva production [37]. In our study the subjects played the tasks only once. In addition, the sound pressure level in the tasks with vapor could not be measured, because the vapor changes the sound characteristics of the instruments. Still, it can be speculated, that our results represent the maximum dispersion distances for soprano recorders, since we asked the trained players to play as loudly as possible. The dynamic range of recorders is limited, because excessively high exhalation flow rates distort the instrument’s sound and pitch [38]. Therefore, it is expected that in usual music practice the measured distances will not be exceeded.

It should also be noted here that the aerosol dispersion reflects only a part of the potential virus transmission pathway which includes (1) the aerosol generation, (2) the aerosol dispersion, (3) the accumulation in closed rooms, and (4) the inhalation of aerosols by the recipient [39]. No aerosol concentrations were measured in the presented study. However, the mechanism and construction of the recorder could be assumed to be comparable to those of flutes and clarinets. Consequently, in application of the data by He et al. [17] also the recorder could be classified in an intermediate risk category. Adding to this estimation, the recorder is mostly played by minors and female adults [21], who are assumed to emit less respiratory aerosols than male adults [35]. This assumption should be verified in future investigations.

The installation of the extended mask and bell hole tissue, covering all holes of the instrument, did not show a clear pattern in comparison to the task without mask (T3), with values evenly distributed to higher, lower, and comparable categories. Although the median values to the front were lower with the safety procedure, the maxima were higher, reaching 2.28 m after ten seconds demonstrating a greater variance among all measurements.

Playing with a paper towel closing the bell hole tightly showed many values that were even higher than without it, i. e., 9 out of 12 values in x- and y direction (see Table 1). This might be due to pressure-supercompensation to the perceived resistance at the end of the tube, augmenting the jet stream at the narrow labium. The maximum side diameter of the cloud reached 1.29 m 10 seconds after the end of the task. Consequently, a covering of the bell hole without covering the labium is considered worse than playing without any device. These results are in agreement with Volckens et al. [33] and Firle et al. [34], who did not find aerosol emissions reduced by bell covers in woodwind instruments. A proper fit of coverings has been showed to be crucial for protection effectiveness [40]. However, this was found to be difficult for recorders, since, according to the trained players of our study and also reported by Firle et al. [34], it impaired the sound and further the free movement. The bell hole tissue was rated to be feasible, but was also not effective in reducing the dispersion distance. Sound attenuation was not visible in the LTAS of any of the installations, but a tight covering of the bell hole prohibited the playing of low pitches where all finger holes are closed. The suction funnel was the only safety installation that showed convincing effects in all directions at all time points. However, the device needs significant technical effort to be installed correctly while producing as little noise as possible so as not to disturb the music, which could make teachers and institutions hesitant. Thus, none of the evaluated potential safety measures fulfilled all the needs of the recorder players and the function as protection mechanism.

### Limitations

The presented study concentrated on the impulse dispersion of a single musical phrase. However, in real life situations, the rehearsal of a whole piece takes much longer and, consequently, aerosol clouds could accumulate in the room. Additionally, the study measured motionless, single players. It can be expected that several people playing together in classes or groups will produce complex flows in the room [14], which underlines the importance of aerating the room frequently or with opposing windows open. Furthermore, the measured values represent the distance reached by the aerosol cloud but not the number of aerosols or their concentration in the cloud. Additionally, the potential virus dose in such a cloud differs by subject and in the course of disease. Still, the data from this study can help to assess where direct steady aerosol streams are likely to be found while playing and therefore can be avoided. Another limitation of this study is the small sample size of 5 subjects, which prevents statistical analyses and generalizability and can therefore only provide a first impression of the matter.

## Conclusion

This study investigated the impulse dispersion of aerosols in soprano recorders and evaluated three different potential safety devices concerning the spread of aerosols, i. e., different types of covering of the holes and a suction funnel. The pure aerosol cloud dispersion was found to be comparable to those of the clarinet measured in a previous study. Concerning the evaluated potential safety installations, the different types of covering of the instrument did not show clear benefits in prohibiting the expansion of the aerosol clouds and were furthermore rated as impracticable by the subjects. The suction funnel showed almost total reduction of the aerosol clouds and, thus, seems to be an effective method to remove the aerosol particles immediately after the emission from the instrument.

## Supporting information

S1 File Questionnaire for subjective evaluation of the potential safety devices. (PDF)
