# Supplementary material for: Impulse dispersion of aerosols during playing the recorder and evaluation of safety measures
Source: PLoS One. 2022 Sep 26;17(9):e0266991. doi: 10.1371/journal.pone.0266991 (PMC9512216; doi:10.1371/journal.pone.0266991)
Supplement: S1 File — (PDF) [file pone.0266991.s001.pdf]

S1: Questionnaire for subjective evaluation of the potential safety devices

Schutzvorrichtung *[protective device]:* ____________________

1. Ist die **Bewegungsfreiheit** eingeschränkt, sodass das Musizieren beeinträchtigt wird?

   *[Is **freedom of movement** for making music impaired?]*

Trifft gar nicht zu *[Does not apply]*		trifft sehr zu *[Applies]*

2. Kann ausreichend und schnell **eingeatmet** werden?

   *[Is quick and sufficient **inhalation** possible?]*

Trifft gar nicht zu		trifft sehr zu

3. Tritt das Gefühl auf, dass der Schall **gedämpft** übertragen wird?

   *[Does sound transmission seem to be **attenuated**?]*

Trifft gar nicht zu		trifft sehr zu

4. Ich kann mir vorstellen, mit dieser Schutzvorrichtung Konzerte zu bestreiten.

   [I can imagine playing concerts with this protective device.]

Nein	Ja

5. [No| Yes]

ggf. Kommentar: *[possible comment:]*____
____
